# Operation of Lithotrity

**Published:** 1843-11

**Authors:** William A. McDowell

**Affiliations:** Louisville


Art. II.—*Operation of Lithotrity.* By William A. McDowell, M.D., of Louisville.

A detail of the following operation is offered to the profession, chiefly on account of the advantage derived from the co-operation of the *slippery-elm* bougie; an instrument which, for many years past, I have employed in the treatment of strictures, with a greater degree of success than I have ever seen ascribed to any other method.

J. V., aged eighteen, well grown, and of good constitution, who had suffered from symptoms of stone since his earliest recollection, was sounded August 17th, 1843. The sound indicated a plurality of stones—emitting the click right and left when twirled to either side. Prescribed decoction of mallows for constant drink; bowels to be kept loose with crem. tart.; moderate and unstimulating diet, preparatory to the operation of lithotrity.

The sound used, measures within a fraction of four lines in circumference; it passed the prostate gland with difficulty, and gave more pain than any subsequent step in the operation. To remedy this condition, three graduated bougies of seasoned slippery-elm bark were ordered, the first about the size of the sound that had been used. These, after immersion for a few minutes in tepid water, were successively, in the course of ten days, introduced through the urethra, and there suffered to remain and expand by imbibing the moisture incident to the situation. The last of the number, expanding to a circumference exceeding thirteen lines, afforded space for the easy introduction and free use of a lithotritor (Heurteloup's) of a circumference of eleven lines.

The operation was commenced August 30th, in presence of Drs. Donne and Bayless. The first grasp of the lithotritor embraced a stone which the scale of the instrument indicated to be of the diameter of nine lines. This slipped from the instrument. A second stone measuring twelve lines, was seized and broken to pieces. Five fragments were afterwards grasped and crushed; this completed the first sitting. Within the succeeding twenty-four hours, a large quantity of calculous matter was discharged in powder and fragments.

September 2d. At this the second sitting, twelve fragments, of diameters varying from three to six lines, were crushed; which was followed by a copious discharge of calculi. The calculous substance discharged after these sittings, was of a reddish or Spanish-brown colour, hard, but of inferior gravity. Much of the matter (all of the powdered) was lost through the patient's want of skill in collecting it; the exact quantity is therefore not noted.

September 4th. Patient considers himself cured, having experienced no symptom of stone for two days; but a calculus is yet detected by the sound.

Third sitting. A stone of nine lines diameter was seized; it proved to be extremely solid, and difficult to break down. Eight subsequent crushes of fragments completed the sitting. Calculi to the amount of fifty-nine grains were discharged within the succeeding twenty-four hours; the largest fragment measured eight lines in length, and five in breadth, from point to point of opposite angular projections. This produced some degree of irritation in the urethra, on account of which the next sitting was deferred to September 16th.

September 16th: fourth sitting. Sixteen fragments varying from three to six lines in diameter were crushed; seventy-seven grains of calculous matter were discharged within twenty-four hours thereafter. Since which, I have explored the bladder by various successive soundings, and on the 20th sounding was performed by Professor Gross, and by Mr. A. Martin, late house-surgeon at the Louisville Marine Hospital. No indication of any remaining calculi having been discovered, the patient was dismissed cured.

Mr. V. thinks he suffered no more pain during the process than he had ordinarily from the stone alone. He walked to my office, a distance of nearly a mile, to each sitting, and immediately after returned in the same way. He never lost a meal, nor was confined an hour, but considered himself as able to perform his ordinary avocations during the process as he ever had been.

*October*, 1843.

